# Forced turnover of aged microglia induces an intermediate phenotype but does not rebalance CNS environmental cues driving priming to immune challenge

**DOI:** 10.1186/s40478-018-0636-8

**Published:** 2018-11-26

**Authors:** Shane M. O’Neil, Kristina G. Witcher, Daniel B. McKim, Jonathan P. Godbout

**Affiliations:** 1 Department of Neuroscience, The Ohio State University Wexner Medical Center, Columbus, OH USA; 2 Institute for Behavioral Medicine Research, The Ohio State University Wexner Medical Center, 231 IBMR Building, 460 Medical Center Drive, Columbus, OH 43210 USA; 3 Chronic Brain Injury Program, The Ohio State University, Columbus, OH USA

**Keywords:** Microglia, Age, Priming, CSF1R antagonist, Lipopolysaccharide, RNA-Seq

## Abstract

Microglia are the resident innate immune cells of the central nervous system. Limited turnover throughout the lifespan leaves microglia susceptible to age-associated dysfunction. Indeed, we and others have reported microglia develop a pro-inflammatory or “primed” profile with age, characterized by increased expression of inflammatory mediators (e.g., MHC-II, CD68, IL-1β). Moreover, immune challenge with lipopolysaccharide (LPS) causes an exaggerated and prolonged neuroinflammatory response mediated by primed microglia in the aged brain. Recent studies show colony-stimulating factor 1 receptor (CSF1R) antagonism results in rapid depletion of microglia without significant complications. Therefore, we hypothesized that CSF1R antagonist-mediated depletion of microglia in the aged brain would result in repopulation with new and unprimed microglia. Here we provide novel evidence that microglia in the brain of adult (6–8 weeks old) and aged (16–18 months old) BALB/c mice were depleted following 3-week oral PLX5622 administration. When CSF1R antagonism was stopped, microglia repopulated equally in the adult and aged brain. Microglial depletion and repopulation reversed age-associated increases in microglial CD68^+^ lysosome enlargement and lipofuscin accumulation. Microglia-specific RNA sequencing revealed 511 differentially expressed genes with age. Of these, 117 genes were reversed by microglial repopulation (e.g., *Apoe*, *Tgfb2*, *Socs3*). Nevertheless, LPS challenge still induced an exaggerated microglial inflammatory response in the aged brain compared to adults. RNA sequencing of whole-brain tissue revealed an age-induced inflammatory signature, including reactive astrocytes, that was not restored by microglial depletion and repopulation. Furthermore, the microenvironment of the aged brain produced soluble factors that influenced developing microglia ex vivo and induced a profile primed to LPS challenge. Thus, the aged brain microenvironment promotes microglial priming despite repopulation of new microglia. Collectively, aged microglia proliferate and repopulate the brain, but these new cells still adopt a pro-inflammatory profile in the aged brain.

**Electronic supplementary material:**

The online version of this article (10.1186/s40478-018-0636-8) contains supplementary material, which is available to authorized users.

## Introduction

Aging robustly affects the bidirectional communication between the brain and immune system [14, 35]. This essential communication involves microglia and astrocytes, which interpret inflammatory signals from the periphery and propagate them within the central nervous system (CNS) [17, 29, 44, 46]. Moreover, central inflammatory signaling is critical for normal physiological and behavioral responses to infection [16]. With aging, this altered neuro-immune communication results in heightened risk of mortality and co-morbidity of depression or dementia [34, 36, 51, 53, 58]. For instance, acute bacterial infection in elderly patients often presents as acute cognitive impairment and altered mood [2, 19]. Moreover, these individuals are at an increased risk for progressive dementia and cognitive impairment even after the infection resolves [33]. These data are consistent with rodent studies showing acute immune challenge triggers prolonged neuroinflammatory responses, altering affective behavior and cognition [17, 28]. For instance, immune challenge by lipopolysaccharide (LPS) or *Escherichia coli* in aged rodents induces elevated neuroinflammation, prolonged sickness behavior, and acute cognitive impairment, which are attributed to activation of microglia and astrocytes [5, 6, 26, 27, 73]. In humans, these infection-related neurological and psychiatric complications reduce both quality of life and life expectancy [51, 52, 62, 67, 69]. Therefore, understanding how aging impacts glial interactions in the brain and thereby leads to cognitive impairment is of paramount importance.

There is evidence that microglia and astrocytes develop a more pro-inflammatory or “primed” profile as a result of normal aging [47]. For instance, microglia in the aged brain have increased expression of several inflammatory markers, including major histocompatibility complex (MHC) class II proteins, and adopt a de-ramified morphology with thicker processes [13, 24, 29, 32, 56, 61, 63, 64, 68]. Additionally, astrocytes in the aged brain have increased baseline levels of glial fibrillary acidic protein (GFAP) and vimentin, both of which indicate increased reactivity [15, 26, 42]. While the presence of these primed glia is insufficient to induce cognitive dysfunction, primed glia mediate exaggerated and prolonged neuroinflammatory responses to peripheral immune challenge. This hyper-inflammatory response in the CNS is not mirrored by the peripheral innate immune response, which is intact in aged animals [4, 12, 14, 26, 29, 73]. Indeed, when the CNS is stimulated directly with intracerebroventricular (i.c.v.) LPS or gp120, aged mice still exhibit an exaggerated and prolonged sickness responses [1, 31]. Thus, aged glia adopt a primed profile with age, leaving the elderly susceptible to hyper-inflammatory CNS reactions to acute peripheral stimuli.

Recent studies show that microglia can be depleted from the rodent CNS through colony-stimulating factor 1 receptor (CSF1R) antagonism without significant complications [20, 55]. Moreover, cessation of this antagonism results in rapid microglial repopulation. Rice et al. (2017) used this approach to promote microglial turnover following inducible hippocampal neuron death and found microglial depletion and repopulation following hippocampal lesion ameliorated chronic microgliosis, leukocyte infiltration, and inflammatory gene expression [55]. Furthermore, this was associated with improved cognitive and behavioral recovery. Recently, Elmore et al. (2018) found that depletion and repopulation of microglia in aged mice restored age-associated changes in microglial morphology [21]. This was associated with a reversal of age-associated hippocampal dendritic spine loss and cognitive decline. Thus, depletion and repopulation of microglia may present a therapeutic strategy for redirecting chronic microglia-mediated inflammation.

The purpose of this study was to determine the degree to which CSF1R antagonist-mediated depletion of microglia in the aged brain would result in repopulation with new and unprimed microglia. Here, we provide novel evidence that promoting forced turnover of aged microglia reduced intracellular accumulation of lipofuscin and restored lysosome size to adult levels. While repopulated microglia in the aged brain had an intermediate RNA signature compared to aged controls, they remained primed to peripheral immune challenge and were hyper-inflammatory when activated. Moreover, age-associated reactive astrogliosis persisted independent of microglial turnover and ex vivo data shows the aged CNS microenvironment promotes microglial priming in neonatal microglia.

## Materials & methods

### Mice and PLX5622 administration

All procedures were performed in accordance with the National Institute of Health Guide for the Care and Use of Laboratory Animals and were approved by The Ohio State University Institutional Animal Care and Use Committee. PLX5622 was provided by Plexxikon (Berkeley, CA) and formulated in standard rodent chow by Research Diets (New Brunswick, NJ) at 1200 mg PLX5622/kg chow. Adult (6–8 weeks old) and aged (16–18 months old) male BALB/c mice from Charles River (Wilmington, MA) were provided vehicle or PLX5622 chow ad libitum for 21 d, after which PLX5622 treatment was withdrawn for 21 d to allow microglia to fully repopulate the CNS. Behavioral testing was performed immediately following this repopulation period.

### Percoll-enrichment of microglia

Microglia were isolated from brain homogenates using a Percoll density gradient as previously described [48]. In brief, mice were sacrificed by CO_2_ asphyxiation. Brains were collected after decapitation and homogenized in ice-cold phosphate-buffered saline (PBS) using a 10 mL Potter-Elvehjem tissue grinder (Wheaton) and the cell pellet resuspended in 70% isotonic Percoll (GE Healthcare). A discontinuous Percoll density gradient was layered and centrifuged at 2000×*g* for 20 min. Enriched microglia were collected from the interface between the 70 and 50% Percoll layers. Of the cells collected from this interface, > 80% of the cells were CD11b^+^/CD45^low^ microglia.

### Microglial lipofuscin detection by flow cytometry

Percoll-enriched microglia were incubated with anti-mouse CD11b-PE-Cyanine7 and CD45-PerCP-Cyanine5.5 antibodies (1:50; eBioscience). Expression was determined using a BD FACSCalibur cytometer. Microglia were identified by CD11b^+^/CD45^low^ expression. Lipofuscin was detected at 488 nm excitation and 515–545 nm emission. Flow data were analyzed using FlowJo software (Tree Star).

### NanoString nCounter analysis of mRNA copy number

Mice were sacrificed by CO_2_ asphyxiation and the hippocampus was dissected and snap frozen in liquid nitrogen (− 196 °C). Hippocampal RNA was isolated using the Tri-Reagent protocol (Sigma-Aldrich). RNA quality and integrity was determined using the Agilent 2200 TapeStation assay (Agilent Technologies). nCounter analysis (NanoString Technologies) was performed by the OSU Comprehensive Cancer Center (OSUCCC) Genomics Shared Resource facility (The Ohio State University, Columbus, OH) using the Mouse Inflammation v2 Panel for 248 inflammation-related mouse genes, 20 custom genes, and 6 internal reference controls. Copy numbers were normalized using DESeq2 Bioconductor package in R [41].

### Immunohistochemistry for Iba1 and GFAP

Mice were sacrificed by CO_2_ asphyxiation and transcardially perfused with ice-cold PBS (pH 7.4) and 4% paraformaldehyde (PFA). Brains were collected and post-fixed in 4% PFA for 24 h, cryoprotected in 30% sucrose for 24 h, frozen using dry ice-cooled isopentane (− 78 °C), sectioned coronally at 30 μm on a Leica CM1800 cryostat, and stored in cryoprotectant (30% ethylene glycol, 30% polyethylene glycol, 40% 0.2 M phosphate buffer) for immunolabeling. Next, sections (Bregma - 1.5 mm) were washed in PBS, blocked (5% normal donkey serum, 0.1% Triton X-100, 1% bovine serum albumin in PBS) for 1 h, and incubated with rabbit anti-mouse Iba1 (1:1000; Wako Chemicals) or rabbit anti-mouse GFAP antibody (1:500; Abcam) overnight at 4 °C. Next, sections were washed in PBS and incubated with a fluorochrome-conjugated secondary antibody (Alexa Fluor 594 or Alexa Fluor 488). Sections were mounted on slides and cover-slipped with Fluoromount-G (Invitrogen). Slides were then imaged using a Leica DM5000 B epifluorescent microscope at 20X magnification and captured using a Leica DFC300 FX camera and imaging software. For each animal, 2–4 images were quantified and averaged, and these values were used to calculate the mean and standard error for each experimental group.

### Immunohistochemistry for lipofuscin and CD68

To visualize microglia and neurons, sections (Bregma - 1.5 mm) were incubated with rabbit anti-Iba1 (1:1000; Wako) or rabbit anti-NeuN (1:1000; Millipore) primary antibody, respectively, followed by Alexa Fluor 647 donkey anti-rabbit IgG secondary antibody (1:500, Invitrogen). In order to visualize microglial lysosomes, sections were incubated with rabbit anti-Iba1 (1:1000; Wako) and rat anti-CD68 (1:500; Abcam) primary antibodies, Alexa Fluor 594 donkey anti-rabbit IgG and Alexa Fluor 647 donkey anti-rat IgG secondary antibodies (1:500, Invitrogen), and 0.1% Sudan Black B (Sigma-Aldrich) solution in 70% ethanol for 2 min prior to cover-slipping. Slides were then imaged using a Leica SP8 upright confocal microscope at 63X magnification and sequential optical sections captured using the Leica Application Suite X imaging software. Lipofuscin was imaged at 488 nm excitation and 495–545 nm emission. Sequential optical sections were analyzed using ImageJ software (NIH). For each animal, 2–4 images were quantified and averaged, and these values were used to calculate the mean and standard error for each experimental group.

### RNA sequencing of sorted microglia and coronal brain section

Percoll-enriched microglia were sorted using a Becton-Dickinson FACSAria III cell sorter at the OSUCCC Analytical Cytometry facility. Microglia were identified by CD11b^+^/CD45^low^ expression. Cells were pelleted and lysed in Arcturus PicoPure Extraction Buffer immediately after sorting. The Arcturus PicoPure RNA Isolation Kit (Applied Biosystems) was used to purify and concentrate total RNA from sorted microglia. During RNA isolation, samples were treated with on-column DNase digestion for 15 min at 23 °C to eliminate contaminating genomic DNA. A 1-mm coronal brain section was also collected from each brain and snap frozen in liquid nitrogen. RNA was isolated using the Tri-Reagent protocol (Sigma-Aldrich). RNA quality and integrity was determined using the Agilent 2200 TapeStation assay (Agilent Technologies). RNA-Seq was performed on sorted microglia and brain section (Bregma - 1.5 mm) RNA at the Hussman Institute for Human Genomics Sequencing Core Facility (University of Miami, Miami, FL). Briefly, RNA-Seq libraries were prepared using the Ovation SoLo RNA-Seq System with AnyDeplete rRNA to remove rRNA and other abundant transcripts according to the manufacturer’s recommendation (Nugen). RNA-Seq libraries were run on an Illumina NextSeq 500 sequencing instrument according to the protocols described by the manufacturer.

### Differential gene expression and pathway analysis of microglial and whole-brain RNA sequencing

FASTQ files were aligned to the mouse mm10 genome using STAR Aligner [18]. Raw counts were normalized and differentially expressed genes were determined using the DESeq2 Bioconductor package in R [41]. Factors of unwanted variation were determined with RUVseq and included in the model for differential expression [57]. *P*-values were adjusted for false discovery rate using the Benjamini-Hochberg procedure [7]. To determine reversal of age effects by microglial repopulation, differentially expressed genes between Aged Control and Adult Control microglia (*P*_*adj*_ < 0.05; absolute fold change > 1.5) and brain section (*P*_*adj*_ < 0.05) samples were determined. Next, these genes were determined to be exacerbated (Aged Repopulation vs. Aged Control: *P* < 0.05; conserved directionality), partially reversed (Aged Repopulation vs. Aged Control: *P* < 0.05; Aged Repopulation vs. Adult Control: *P* < 0.05), or reversed (Aged Repopulation vs. Aged Control: *P* < 0.05; Aged Repopulation vs. Adult Control: *P* ≥ 0.05) by microglial repopulation. Pathway analysis was performed using Ingenuity Pathway Analysis (IPA; QIAGEN) [37] and Protein Analysis through Evolutionary Relationships (PANTHER) gene list analysis [66] on genes with baseMean > 10, *P*_*adj*_ < 0.05, and absolute fold change > 1.5. Heat maps were generated and normalized by gene using ‘pheatmap’ in R. The normalized RNA sequencing data is supplied (Additional file 1).

### Immune challenge with lipopolysaccharide

Adult and aged BALB/c mice received a single intraperitoneal (i.p.) injection of saline or *Escherichia coli* LPS (0.33 mg/kg; serotype 0127:B8; Sigma-Aldrich). This LPS dosage was selected because it elicits a pro-inflammatory cytokine response in the brain resulting in a transient sickness response in adult BALB/c mice without mortality in aged mice [8, 26, 72].

### Social exploratory behavior

Social exploration was determined as a measure of sickness behavior as previously described [26]. In brief, mice were injected (*t* = 0) intraperitoneally with saline or 0.33 mg/kg LPS. At *t* = 4 and 24 h, a novel male juvenile C57BL/6 mouse was introduced into the test subject’s home cage for 5 min. Behavior was recorded and the total duration of time the experimental subject engaged in social investigation of the juvenile (e.g. anogenital sniffing, trailing) was determined.

### Gene expression by qPCR

RNA was isolated from a 1-mm coronal brain section (Bregma - 1.5 mm) using the Tri-Reagent protocol (Sigma-Aldrich). Reverse transcription was performed using the High Capacity cDNA Reverse Transcription Kit (Applied Biosystems) to produce cDNA. Quantitative real-time (q)-PCR was performed using the TaqMan Gene Expression Assay (Applied Biosystems). In brief, experimental cDNA was amplified using qPCR such that a target gene and reference gene (*Gapdh*) were amplified simultaneously using an oligonucleotide probe with a 5′ fluorescent reporter dye (FAM) and 3′ non-fluorescent quencher (NFQ). When *Taq* DNA polymerase synthesizes a new strand and reaches the TaqMan probe, the FAM is cleaved from the NFQ and increases the fluorescent intensity proportional to the amount of amplicon synthesized. Fluorescence was determined using a QuantStudio 3 or 5 Real-Time PCR System (Applied Biosystems). Data were analyzed using the comparative threshold cycle (ΔΔC_T_) method and results are expressed as fold change from a control group.

### CNS-conditioned neonatal microglia culture

Adult (8–10 weeks old) and aged (20 months old) male BALB/c mice were sacrificed by CO_2_ asphyxiation and three 1-mm coronal brain sections (Bregma - 1, 2, and 3 mm) were collected from each mouse. These sections were placed three-per-well onto 0.4-μm nylon mesh inserts in 1.5 mL DMEM/F12 (Millipore) supplemented with 25% horse serum and incubated at 37 °C and 5% CO_2_. Media was refreshed after 3 h and conditioned media (CM) collected after another 24 h. CM was then supplemented with fresh DMEM/F12 containing 10% fetal bovine serum (FBS) at a 1:3 ratio. Control media received identical treatment without contact with brain tissue. Primary microglia cultures were established from the brain of neonatal mice as previously described [22]. In brief, whole brains were isolated from neonatal (post-natal day 1–3) BALB/c mice and digested in 0.25% trypsin EDTA (Gibco) for 15 min at 37 °C. Samples were then pelleted and resuspended in growth medium (20% FBS, 100 U/mL penicillin, 100 U/mL streptomycin, 0.25 μg/mL amphotericin B, and 50 μg/mL gentamicin in DMEM/F12). Pellets were mechanically dissociated by pipetting and filtered through a 70-μm cell strainer. Mixed glia cultures were maintained at 37 °C and 5% CO_2_, and media was replenished every 3–4 days until confluency. Mixed glia cultures were then shaken at 150 rpm on a Forma Orbital Shaker (Thermo) at 37 °C for 3 h. Microglia were harvested from the confluent layer and plated at a density of 1 × 10^4^ cells per well on a 96-well plate. Purity was assessed at > 98% using immunocytochemistry (Iba1^+^/DAPI^+^). After 48 h, microglia were washed twice with DMEM/F12, incubated in adult or aged CM for 24 h, and then stimulated with 1 μg/mL LPS. After 4 h, media was aspirated, and microglial RNA extracted using the RNeasy Plus Mini kit (QIAGEN). Parallel cultures were processed using the CellTiter 96 AQ_ueous_ One Solution Cell Proliferation Assay (Promega) to determine cell viability in adult or aged CM.

### Statistical analysis

Statistical analysis was performed using GraphPad Prism software (San Diego, CA) unless otherwise specified. To determine significant main effects and interactions between variables, two-way analysis of variance (ANOVA) was performed. Comparison between groups was performed using Student’s *t*-test and Bonferroni’s correction for multiple comparisons. A value of *P* < 0.05 was considered statistically significant. All data are presented as mean ± standard error of the mean (SEM).

## Results

### CSF1R antagonism depleted microglia in adult and aged BALB/c mice

**Fig. 1:**
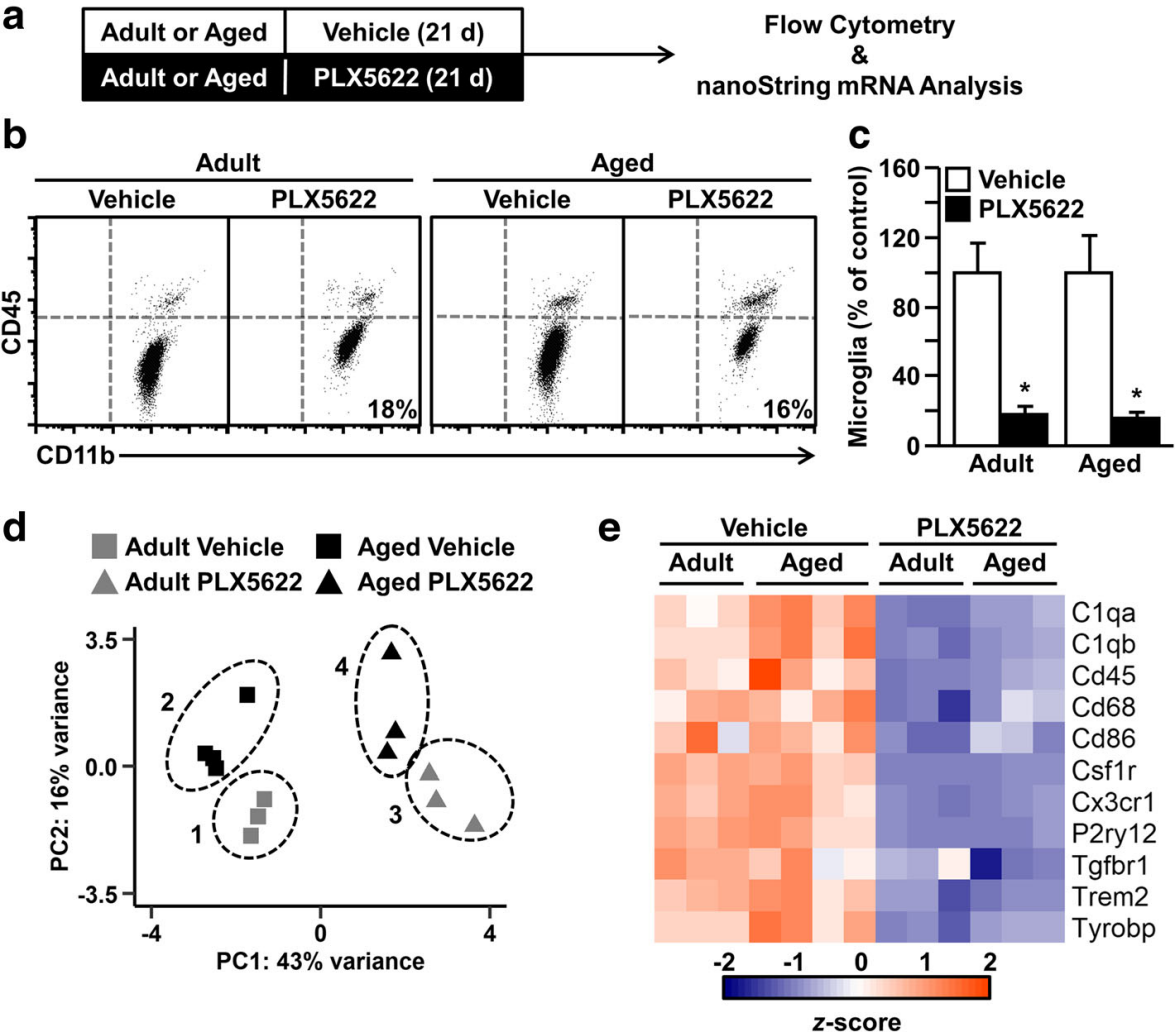
CSF1R antagonism depleted microglia in adult and aged BALB/c mice. **a** Adult (6–8 weeks old) and aged (16–18 months old) male BALB/c mice were provided diets formulated with vehicle or CSF1R antagonist (PLX5622) for 21 d. After 21 d, mice were sacrificed, microglia were Percoll-enriched, and the number of microglia was determined in the brain. **b** Representative bivariate dot plots of CD45 and CD11b labeling of Percoll-enriched microglia. **c** Percent of CD11b^+^/CD45^low^ microglia in the brain of adult and aged mice after 21 days of PLX5622 administration normalized to vehicle controls (*n* = 5 mice / group). In the same mice, the hippocampus was microdissected, and mRNA levels of key microglial signature genes were assessed by nanoString nCounter analysis (*n* = 3–4 mice / group). **d** PCA plot showing unsupervised clustering of treatment groups by both PLX5622 (PC1) and age (PC2). **e** Heat map of hippocampal mRNA signature. Bars represent the mean ± SEM. Means with * are different from age-matched vehicle controls (*P* < 0.05)

We and others report that microglia of the aged brain have a primed and immune-reactive profile [5, 6, 13, 24, 26, 27, 29, 32, 47, 56, 61, 63, 64, 68, 73]. Thus, the objective of this study was to determine if CSF1R antagonist-mediated depletion of microglia in the aged brain would result in repopulation with new and unprimed microglia. First, adult and aged BALB/c mice were administered vehicle or PLX5622 chow for 21 d and the number of microglia (CD11b^+^/CD45^low^) in the brain was assessed (Fig. 1a). Representative bivariate dot plots of CD45 and CD11b labeling of Percoll-enriched microglia are shown (Fig. 1b). As expected, there was a significant main effect of PLX5622 treatment on the number of microglia in the brain (F(1, 16) = 37.11, *P* < 0.0001; Fig. 1c). Moreover, post hoc analysis confirmed PLX5622 administration resulted in an 80–85% reduction in microglia isolated from the brain of adult and aged mice compared to controls (*P* < 0.01, for each). Additionally, RNA was collected from the hippocampus, a region exhibiting profound neuroinflammatory changes in aged rodents [3, 9, 49], and the expression of several microglia-related genes was determined by nanoString nCounter analysis. Principle component analysis (PCA) of hippocampal gene expression shows unsupervised clustering of samples based on age and PLX5622 treatment (Fig. 1d). As expected, expression of key microglial signature genes (e.g., *Cd45*, *Cd68*, *Cd86*, *Csf1r*, *Cx3cr1*, *P2ry12*, *Trem2*) was significantly reduced in mice administered PLX5622 (Fig. 1e). Moreover, this reduction in microglial gene expression with PLX5622 was independent of age. Thus, oral administration of PLX5622 robustly reduced the number of microglia in the brain of both adult and aged BALB/c mice.

### Microglia repopulated independent of age after CSF1R antagonist-mediated depletion

**Fig. 2:**
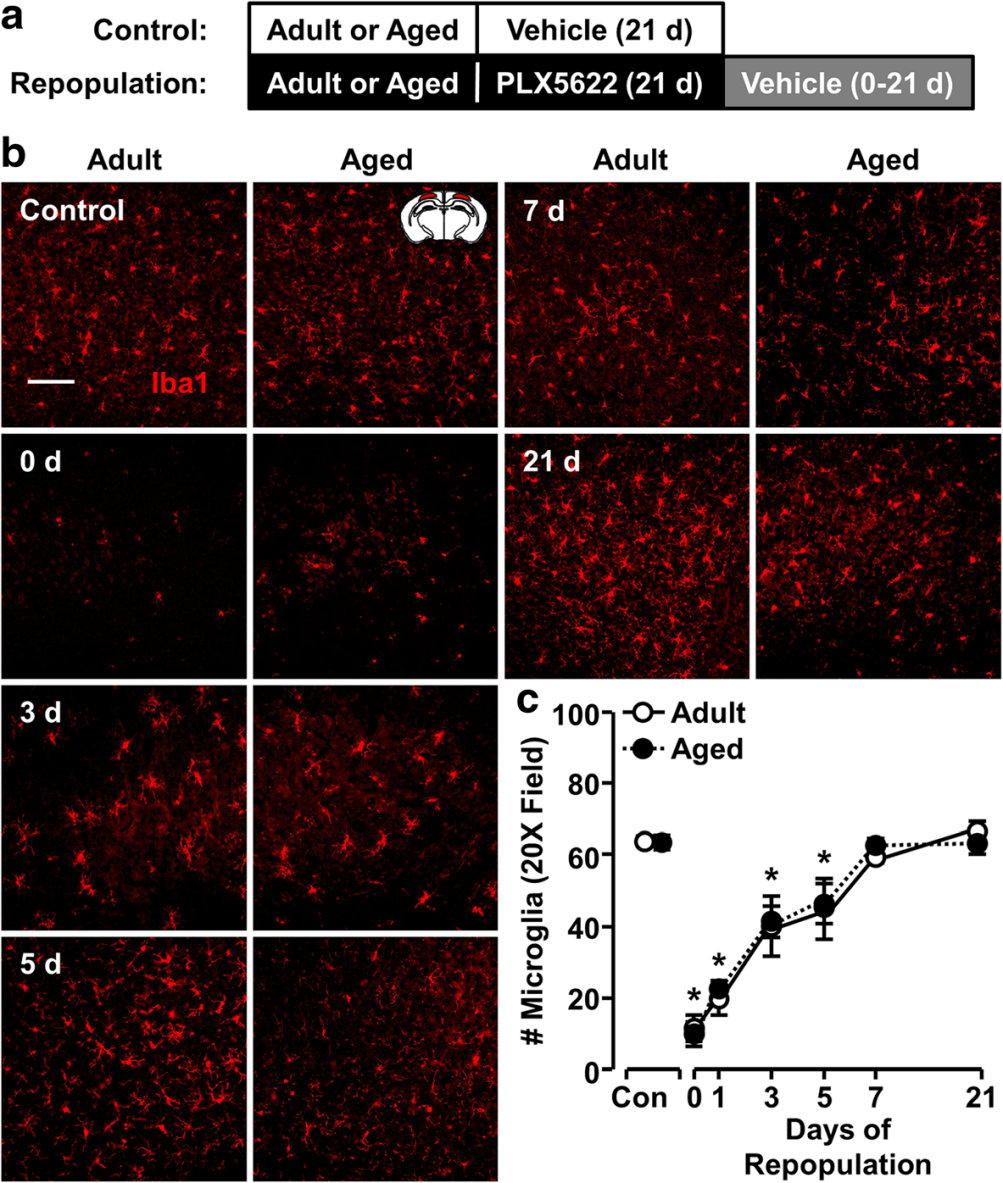
Microglia repopulated independent of age after CSF1R antagonist-mediated depletion. **a** Adult (6–8 weeks old) and aged (16–18 months old) male BALB/c mice were provided diets formulated with vehicle or CSF1R antagonist (PLX5622) for 21 d. After 21 d, all mice were provided vehicle diet for up to 21 d to allow for repopulation of microglia. After 0–21 days of repopulation, the number of microglia was determined in the cortex. **b** Representative Iba1 immunolabeling in the cortex of adult and aged mice at baseline and after 0, 1, 3, 5, 7, and 21 d of repopulation. Scale bar = 100 μm. **c** Number of Iba1^+^ microglia in the cortex at baseline and with 0, 1, 3, 5, 7 and 21 days of repopulation (*n* = 4–7 mice/group). Graphs represent the mean ± SEM. Means with * are different from age-matched controls (*P* < 0.05)

Adult and aged BALB/c mice were administered vehicle or PLX5622 chow for 21 d to deplete microglia. After this, all mice were administered vehicle chow and the level of microglial repopulation was determined 0, 1, 3, 5, 7, or 21 days later (Fig. 2a). Quantification of Iba1^+^ microglia in the cortex showed a main effect of time (F(6, 49) = 42.32, *P* < 0.0001) on microglial repopulation (Fig. 2b, c). For example, after 0 days of repopulation, there was a significant reduction in Iba1^+^ microglia in PLX5622-treated adult and aged mice compared to controls (*P* < 0.0001 for both). In addition, there was a reduced number of microglia after 5 days of repopulation in both adult and aged mice compared to controls. By 7 days, however, microglia returned to control levels in both age groups. Both microglial depopulation and repopulation were independent of age. Overall, the microglia of both adult and aged BALB/c mice were capable of full repopulation after PLX5622-mediated depletion.

### Microglial depletion and repopulation reversed CD68^+^ lysosome size and lipofuscin accumulation in the microglia of aged mice

**Fig. 3:**
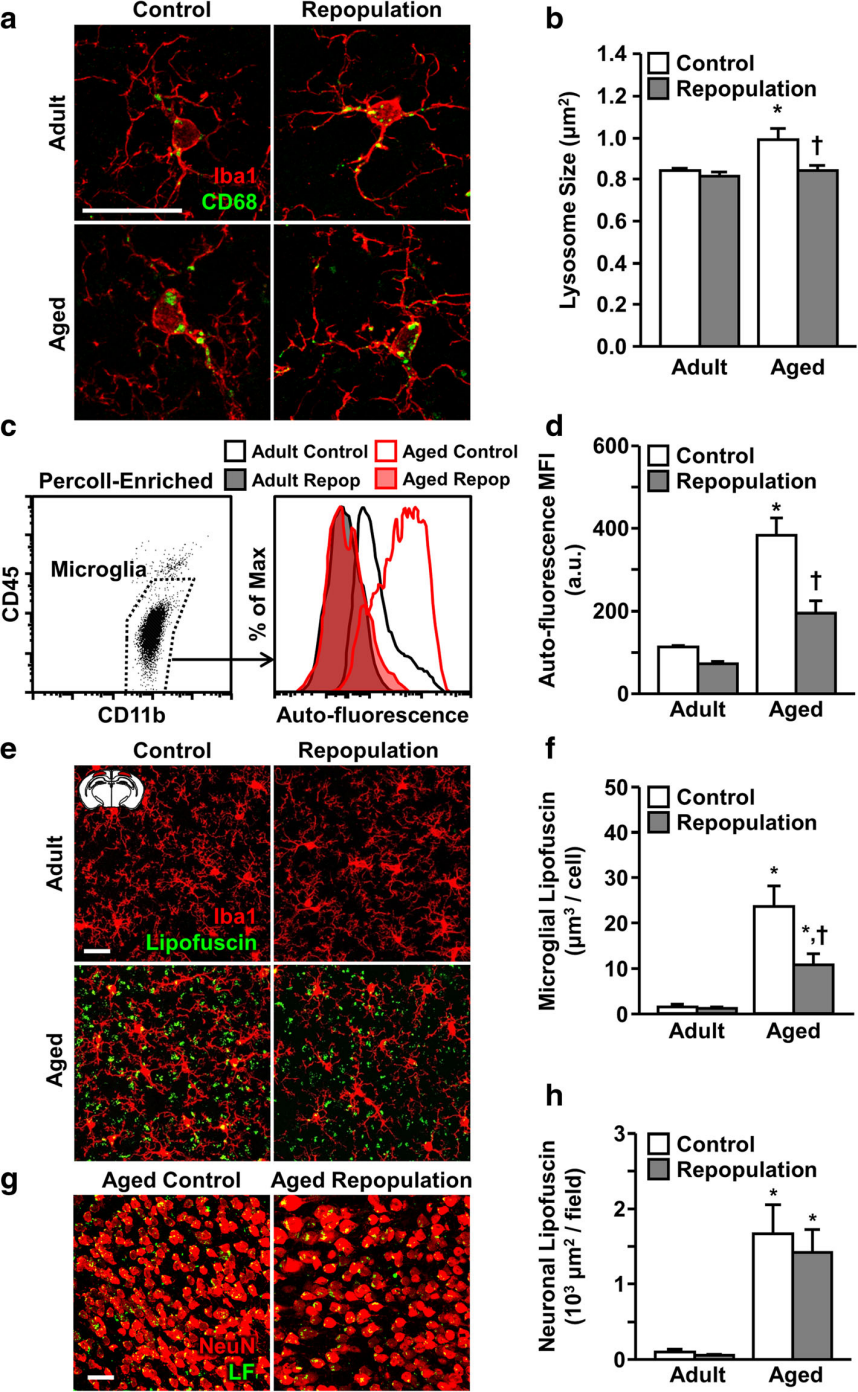
Microglial depletion and repopulation reversed CD68^+^ lysosome size and lipofuscin accumulation in microglia of aged mice. Adult (6–8 weeks old) and aged (16–18 months old) male BALB/c mice were provided diets formulated with vehicle or CSF1R antagonist (PLX5622) for 21 d. After 21 d, all mice were provided vehicle diet for an additional 21 d to allow for repopulation of microglia (Repop). After 21 d of repopulation, CD68 and lipofuscin were assessed in microglia. **a** Representative confocal images of CD68 and Iba1 immunolabeling in the cortex. **b** Quantification of microglial CD68^+^ lysosome size (*n* = 8 mice/group). **c** Representative histograms of auto-fluorescence intensity of CD11b^+^/CD45^low^ microglia by flow cytometry. **d** Mean fluorescence intensity (MFI) of microglial auto-fluorescence by flow cytometry (*n* = 14). **e** Representative confocal images of lipofuscin and Iba1 immunolabeling in the cortex. **f** Quantification of cortical microglial lipofuscin (*n* = 8 mice/group). **g** Representative images of neuronal lipofuscin in the aged brain with and without microglial repopulation. Quantification of neuronal microglial lipofuscin (*n* = 8 mice/group). **h** Quantification of neuronal microglial lipofuscin (*n* = 8 mice/group). Inset shows brain region used for imaging. Scale bars = 25 μm. Bars represent the mean ± SEM. Means with * are different from Adult Control group (*P* < 0.05), and means with † are different from age-matched controls (*P* < 0.05).

Hallmarks of aging include increased CD68 expression and increased accumulation of lipid debris (i.e., lipofuscin) in microglia [60]. CD68 is a lysosomal protein associated with increased macrophage phagocytosis and lipid accumulation. In addition, lipofuscin accumulates in cells over time and is associated with increased cellular auto-fluorescence with age [10, 54]. Thus, we next determined the levels of CD68 and lipofuscin in the microglia of adult and aged mice after microglial depletion and repopulation as described above. A repopulation period of 21 d was selected to avoid confounds of transient microglial activation and cellular debris after PLX5622. The cortex was used for image analysis to minimize confounds of neuronal lipofuscin accumulation. CD68 expression in microglia was relatively abundant in the brain, and there was a significant main effect of age (F(1, 32) = 7.727, *P* < 0.01) and repopulation (F(1, 32) = 8.448, *P* < 0.01) on microglial lysosome size (Fig. 3a, b). Post hoc analysis revealed CD68^+^ lysosome size was increased in cortical microglia of aged mice compared to adults (*P* < 0.01). Moreover, microglial repopulation attenuated this age-associated lysosome enlargement (*P* < 0.01). These data indicate microglial depletion and repopulation normalizes the increased CD68 expression in aged microglia to adult levels.

Next, auto-fluorescence was assessed in microglia after depletion and repopulation. Representative histograms of auto-fluorescence detected in microglia (CD11b^+^/CD45^low^) enriched from the brains of adult and aged mice with or without forced turnover show different distributions of auto-fluorescence between groups (Fig. 3c). As expected, there was a significant main effect of age (F(1, 49) = 58.79, *P* < 0.0001) and repopulation (F(1, 49) = 20.56, *P* < 0.0001) on microglial auto-fluorescence (Fig. 3c). Furthermore, there was a significant interaction between age and repopulation (F(1, 49) = 8.14, *P* < 0.01). Post hoc analysis confirmed microglia from aged mice had higher auto-fluorescence compared to adult controls (*P* < 0.0001). Moreover, this age-associated increase was attenuated in aged mice subjected to microglial depletion and repopulation (*P* < 0.0001).

In a related study, lipofuscin volume was determined in cortical microglia after depletion and repopulation. Notably, there was robust lipofuscin auto-fluorescence in the cortex of aged mice, but minimal auto-fluorescence in the cortex of adult mice (Fig. 3e, f). There was a significant main effect of age (F(1, 32) = 47.41, *P* < 0.0001) and microglial repopulation (F(1, 32) = 8.35, *P* < 0.01) on microglial lipofuscin volume. Furthermore, there was a significant interaction between age and repopulation (F(1, 32) = 7.403, *P* < 0.05). Post hoc analysis confirmed microglial lipofuscin content was higher in aged mice compared to adult controls (*P* < 0.0001), and this age-associated lipofuscin accumulation in microglia was reduced by microglial depletion and repopulation (*P* < 0.01). There was also an age-associated increase in non-microglial lipofuscin (Fig. 3e). NeuN immunolabeling showed neuronal accumulation of lipofuscin with age (F(1, 32) = 41.48, *P* < 0.0001), but this was unaffected by microglial depletion and repopulation (Fig. 3g). Taken together, microglial depletion and repopulation reduced the level of lipofuscin in aged microglia, but not in neurons.

### Depletion and repopulation of microglia partially reversed the microglial aging transcriptional signature

**Fig. 4:**
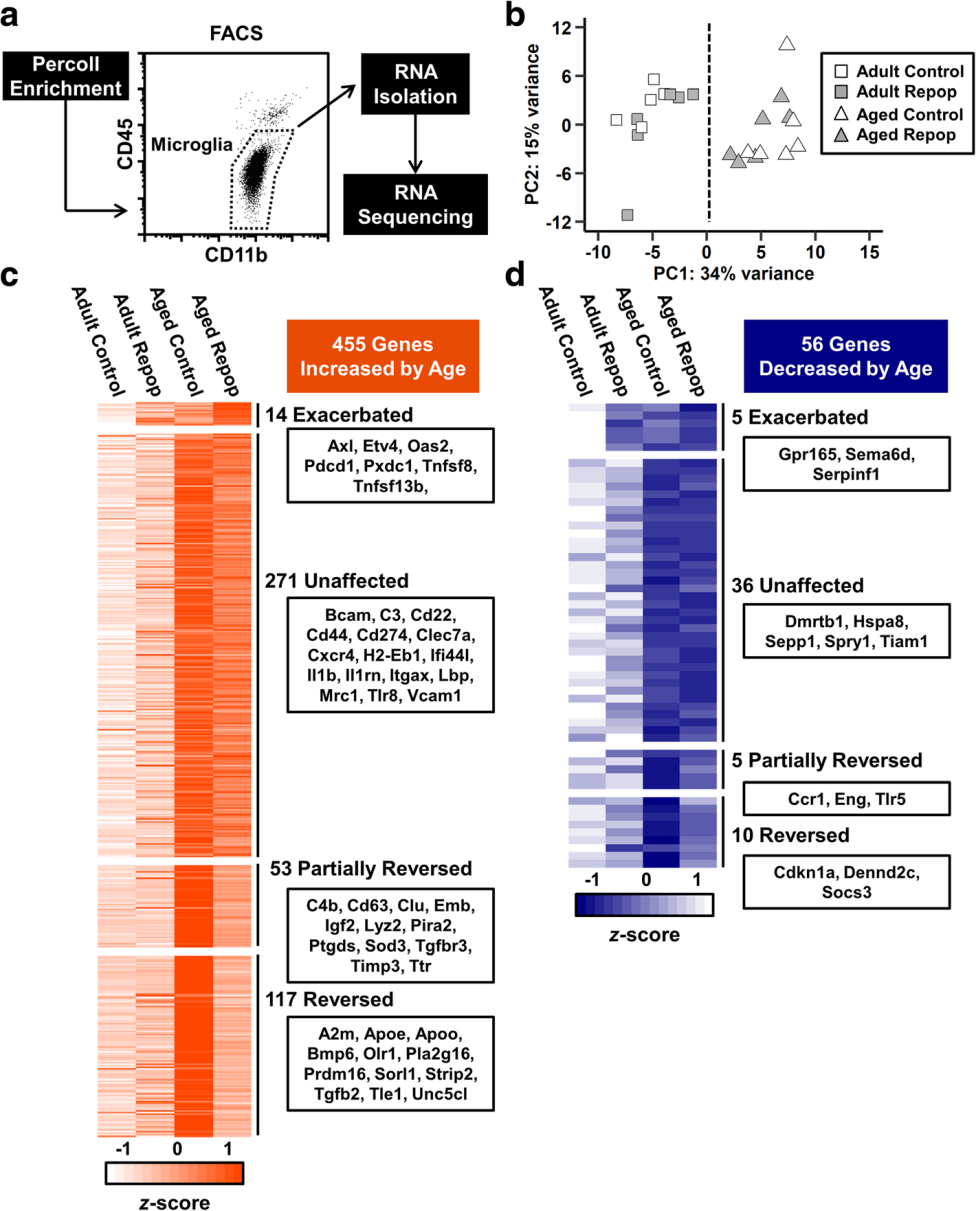
Depletion and repopulation of microglia partially reversed the microglial aging transcriptional signature. **a** Adult (6–8 weeks old) and aged (16–18 months old) male BALB/c mice were provided diets formulated with vehicle or CSF1R antagonist (PLX5622) for 21 d. After 21 d, all mice were provided vehicle diet for an additional 21 d to allow for repopulation of microglia (Repop). After 21 d of repopulation, Percoll-enriched microglia were sorted, and RNA was collected and sequenced. **b** PCA plot shows unsupervised clustering of treatment groups by age (PC1). **c** Heat map shows mean expression of 455 genes significantly increased by age (Aged Control vs. Adult Control: *P*_*adj*_ < 0.05 and fold change > 1.5). These are divided into categories based on patterns of expression following microglial repopulation: Exacerbated (Aged Repop vs. Aged Control: *P* < 0.05; conserved directionality), Partially Reversed (Aged Repop vs. Aged Control: *P* < 0.05; Aged Repop vs. Adult Control: *P* < 0.05), or Reversed (Aged Repop vs. Aged Control: *P* < 0.05; Aged Repop vs. Adult Control: *P* ≥ 0.05). **d** Heat map showing mean expression of 56 genes significantly decreased by age and subdivided as described above. Heat maps are normalized by row and selected genes are shown for each category (*n* = 6 mice/group)

Next, we sought to determine the mRNA signature of microglia in adult and aged mice after depletion and repopulation. Therefore, adult and aged mice were administered vehicle or PLX5622 chow for 21 d to deplete microglia. After 21 d, all mice were administered vehicle chow for an additional 21 d to allow for microglial repopulation. CD11b^+^/CD45^low^ microglia were then Percoll-enriched, purified using fluorescence-activated cell sorting (FACS), and RNA was sequenced (Fig. 4a). PCA on the 500 most variable genes between the experimental groups shows clustering of samples by age, independent of microglial repopulation (Fig. 4b).

Differential expression analysis between Aged Control and Adult Control groups shows 511 genes significantly regulated by age (*P*_*adj*_ < 0.05, absolute fold change > 1.5). Of these, expression of 455 genes was increased (Fig. 4c) while 56 were decreased (Fig. 4d). Following microglial repopulation, age-associated changes in 127 genes were reversed (117 increased, 10 decreased), such that expression in Aged Repopulation was no longer different from Adult Control. For instance, repopulation attenuated age-associated increases in *A2m*, *Apoe*, *Bmp6*, *Olr1*, *Sorl1*, and *Tgfb2* expression. Microglial repopulation also reversed age-associated decreases in expression of *Cdkn1a*, *Dennd2c*, and *Socs3*. Next, gene expression between Aged Repopulation and Aged Control groups were compared to determine genes that were either partially reversed or exacerbated by repopulation (*P* < 0.05). Partially reversed genes showed expression closer to Adult Control, but were not fully restored. In contrast, exacerbated genes were further increased or decreased by repopulation. Age-associated differential expression of 58 genes (53 increased, 5 decreased), was partially reversed by microglial repopulation (Fig. 4c, d). The aged associated increase in *Lyz2* and *Tgfbr3*, and decrease in *Ccr1* and *Tlr5*, were partially reversed by microglial depletion and repopulation. Notably, expression of 19 genes (14 increased, 5 decreased) were exacerbated by microglial repopulation. For instance, age-associated increases in expression of *Axl*, *Oas2*, and *Tnfsf8* were further increased by repopulation (Fig. 4c). Collectively, of the 511 genes differentially regulated by age, 127 were reversed by microglial depletion and repopulation, 53 were partially reversed, and 307 genes (271 increased and 36 decreased) remained unaffected. Genes unaffected by microglial depletion and repopulation included several inflammation-related genes (e.g., *C3*, *Clec7a*, *Ifi44l*, *Il1b*, *Il1rn*, *Mrc1*, *Tlr8*), indicating the age-associated inflammatory profile of microglia was unaffected by forced turnover. Overall, microglia exhibited a robust age-associated mRNA signature that was only partially influenced by forced turnover of microglia.

### Differentially regulated pathways and gene ontologies in microglia were not affected by microglial depletion and repopulation

**Fig. 5:**
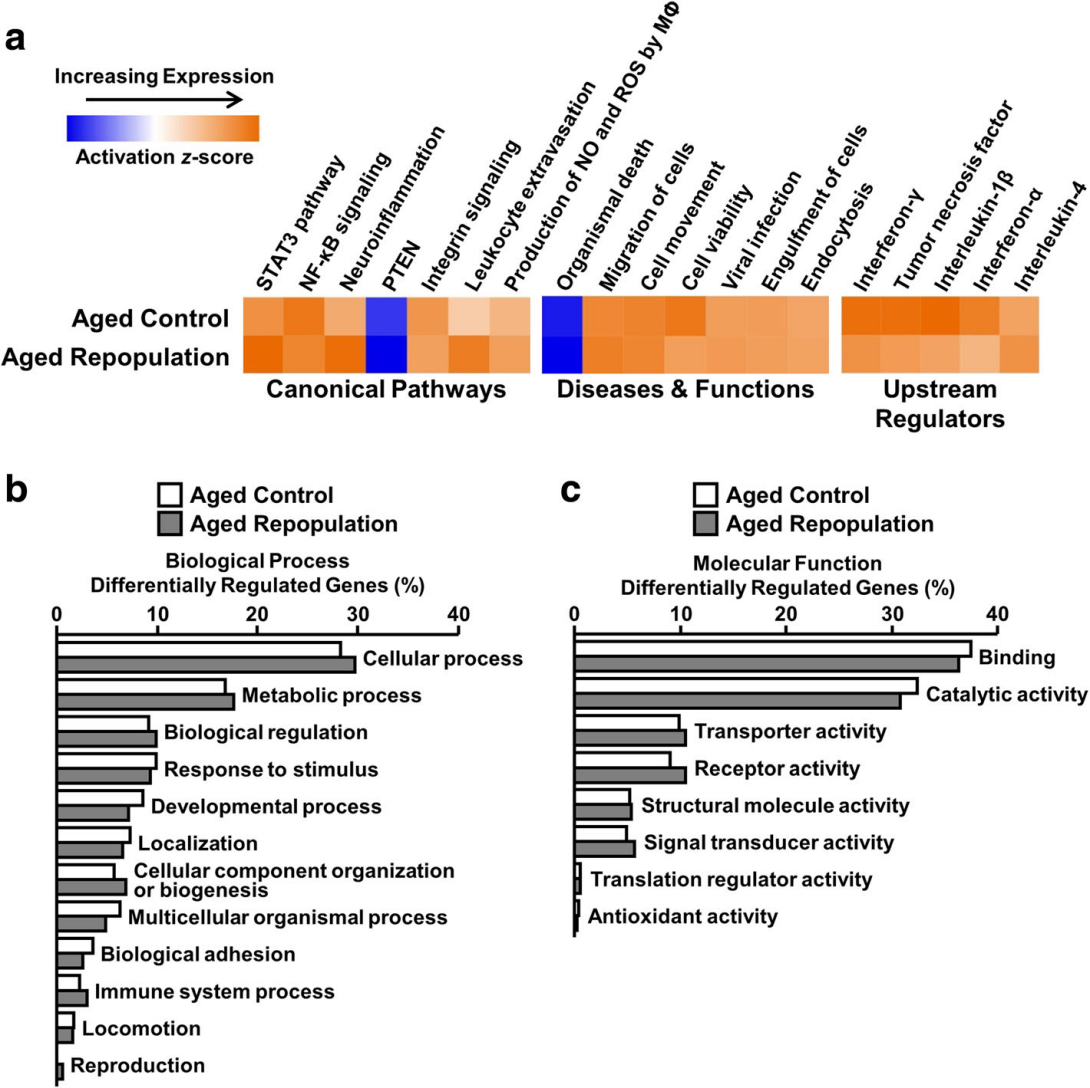
Differentially regulated pathways and gene ontologies were unaffected by microglial depletion and repopulation. Adult (6–8 weeks old) and aged (16–18 months old) male BALB/c mice were provided diets formulated with vehicle or CSF1R antagonist (PLX5622) for 21 d. After 21 d, all mice were provided vehicle diet for an additional 21 d to allow for repopulation of microglia. After 21 d of repopulation, Percoll-enriched microglia were sorted, and RNA was collected and sequenced. Lists of differentially expressed genes were generated by comparing Aged Control and Aged Repop to Adult Controls (baseMean > 10, *P*_*adj*_ < 0.05, and absolute fold change > 1.5) and used for pathway analyses. **a** Heat map of results from IPA Comparison Analysis of Canonical Pathways, Diseases & Functions, and Upstream Regulators performed on genes differentially expressed in the Aged Control and Aged Repopulation groups compared to Adult Control. All pathways shown were significantly regulated by age (*P* < 0.05). **b** Proportions of genes categorized for each GO Biological Process and **c** GO Molecular Function determined by PANTHER Gene Ontology assessment (*n* = 6 mice / group)

The genes differentially expressed by microglia in the Aged Control and Aged Repopulation groups compared to Adult Control (Fig. 4) were analyzed using IPA and PANTHER gene annotation. Canonical pathways, diseases and functions, and upstream regulators that were enriched in each differential expression comparison were compared (Fig. 5a). Overall, there was an age-associated increase in numerous inflammatory pathways, including NF-κb signaling, neuroinflammatory signaling, and production of NO and ROS by macrophages. Furthermore, microglial gene expression in aged mice was consistent with increased signaling of interferon (IFN)-γ, tumor necrosis factor (TNF), interleukin (IL)-1β, IFN-α, and IL-4. Overall, none of these inflammatory pathways were significantly reversed by microglial depletion and repopulation.

Next, PANTHER was used to determine the gene ontology (GO) designations for microglial genes significantly regulated by age with or without repopulation. Genes related to several biological processes (Fig. 5b) and molecular functions (Fig. 5c) were significantly altered by age. The most prevalent biological processes regulated by age in microglia were categorized as cellular process (GO:0009987), metabolic process (GO:0008152), response to stimulus (GO:0050896), biological adhesion (GO:0022610), and biological regulation (GO:0065007). Molecular functions of genes regulated by age included catalytic activity (GO:0003824), binding (GO:0005488), transporter activity (GO:0005215), and receptor activity (GO:0004872). The biological processes and molecular functions of genes regulated by age were unaffected by microglial depletion and repopulation. Taken together, these data indicate that microglial repopulation did not significantly alter the overall pathway-level or cellular systems-level effects of aging on microglial gene expression.

### LPS-induced sickness behavior was prolonged in aged mice and unaffected by microglial depletion and repopulation

**Fig. 6:**
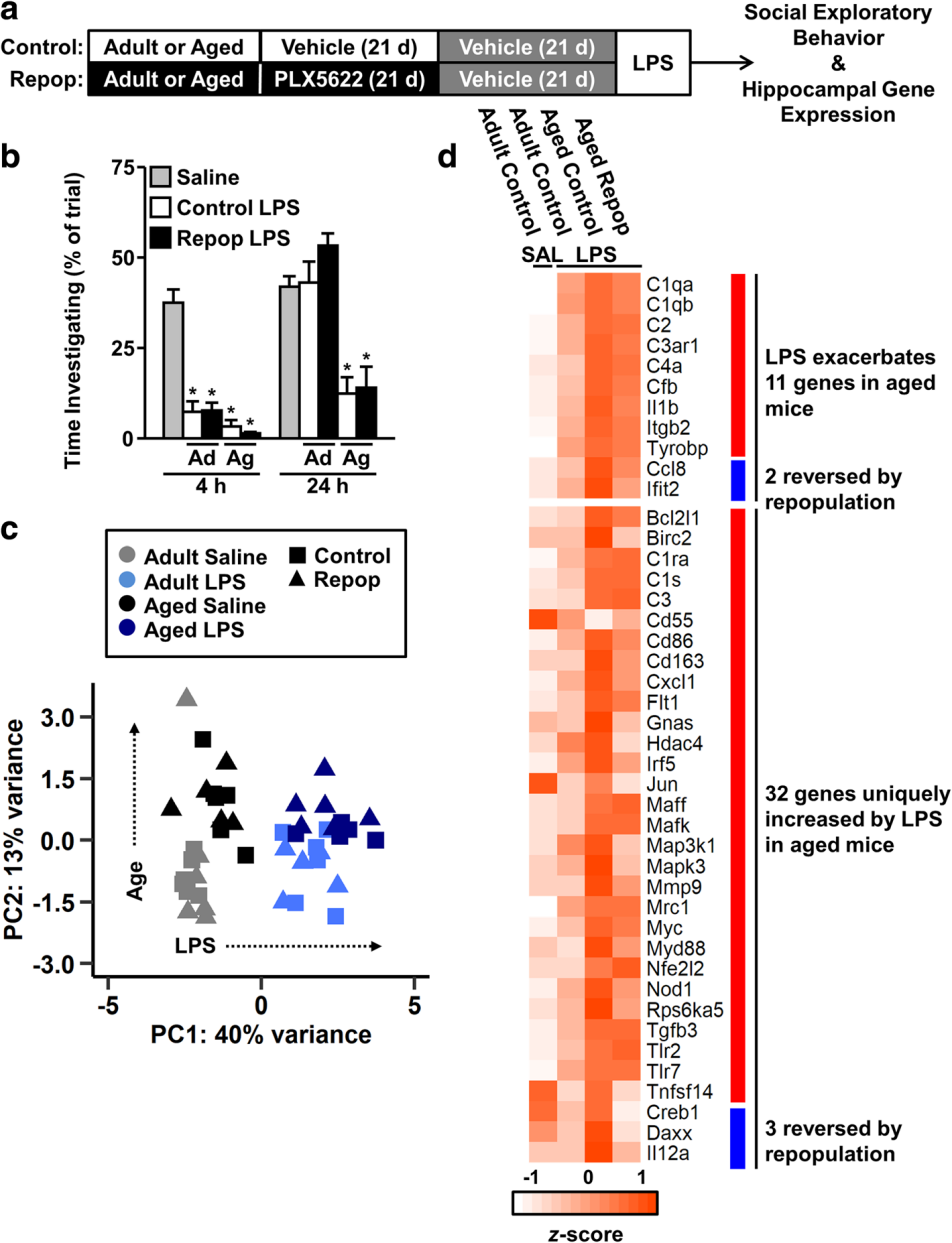
LPS-induced sickness behavior was prolonged in aged mice and unaffected by microglial depletion and repopulation. **a** Adult (6–8 weeks old) and aged (16–18 months old) male BALB/c mice were provided diets formulated with vehicle or CSF1R antagonist (PLX5622) for 21 d. After 21 d, all mice were provided vehicle diet for an additional 21 d to allow for repopulation of microglia (Repop). After 21 d of repopulation, mice were injected with a single dose of i.p. saline or LPS (0.33 mg/kg). **b** Time spent interacting with a novel juvenile mouse 4 and 24 h after LPS or saline administration (*n* = 5–10 mice / group). At 24 h after LPS injection, the hippocampus was microdissected and RNA isolated. NanoString nCounter analysis was used to determine copy number of 248 inflammation-related genes. **c** PCA plot showing unsupervised clustering of groups by LPS (PC1) and age (PC2). **d** Heat map shows average expression of genes differentially expressed between saline-injected Adult Controls and LPS-injected Adult Control, Aged Control, and Aged Repopulation groups. Top cluster reflects genes exacerbated by LPS in aged mice. Bottom cluster reflects genes uniquely increased by LPS in aged mice. Blue annotation highlights genes in which LPS-associated increases were prevented in Aged Repopulation mice (*n* = 5–6 mice/group). Bars represent the mean ± SEM. Means with * are different from Saline (*P* < 0.05)

We and others have reported that primed microglia in models of aging, traumatic brain injury, and stress, exhibit an exaggerated immune-reactive profile after secondary immune challenge [23, 43, 72]. This amplified neuroinflammation corresponds with increased cognitive impairment and prolonged sickness behavior. Therefore, we sought to determine if the intermediate restoration of the microglial mRNA profile corresponded with an attenuated response to peripheral LPS challenge. Thus, adult and aged BALB/c mice were administered vehicle or PLX5622 chow for 21 d to deplete microglia. After 21 d, all mice were administered vehicle chow for an additional 21 d to allow for microglial repopulation, after which mice were injected with i.p. saline or LPS (Fig. 6a). The social exploratory behavior of each mouse was evaluated 4 and 24 h after saline or LPS injection. At both 4 and 24 h post-injection, there was no significant effect of age or repopulation on social exploratory behavior in saline-treated mice; therefore, all saline-treated mice were combined into a single group for subsequent analysis. At 4 h post-injection, there was a significant main effect of LPS (F(1, 46) = 82.16, *P* < 0.001) and age F(1, 20) = 7.56, *P* < 0.05) on social exploratory behavior (Fig. 6b). Post hoc analysis revealed that all LPS-injected mice had decreased social exploratory behavior compared to saline-treated mice (*P* < 0.0001 for all). At 24 h post-injection, there was a main effect of age (F(1, 71) = 37.26, *P* < 0.0001) and LPS (F(1, 71) = 8.39, *P* < 0.01) on social exploratory behavior. Moreover, there was a significant interaction between age and LPS (F(1, 71) = 9.53, *P* < 0.01). Post hoc analysis revealed that only aged LPS-injected mice displayed decreased social exploratory behavior at 24 h compared to saline-treated mice (*P* < 0.0001 for both Control and Repopulation). Within LPS-treated mice, there was a significant main effect of age (F(1, 33) = 47.19, *P* < 0.0001) on social exploration, but no effect of microglial repopulation. Moreover, aged mice spent approximately 70% less time interacting with the novel juvenile compared to adult mice (*P* < 0.001). It is important to note, however, that there was no effect of microglial repopulation on social exploration in either adult or aged mice at either time point. Taken together, aged mice had an exaggerated and prolonged sickness behavior that was not ameliorated by microglial turnover.

To characterize the neuroinflammatory transcriptional response to peripheral immune challenge, hippocampal mRNA was collected 24 h after LPS. RNA was analyzed using the Mouse Inflammation v2 nanoString gene array. PCA showed unsupervised clustering by LPS and age, but not by microglial repopulation (Fig. 6c). Next, LPS-induced changes in inflammatory gene expression were determined in adult and aged mice with or without microglial repopulation (Fig. 6d). Genes were classified as either exacerbated by age (increased by LPS in adults and further increased with age) or uniquely regulated by LPS in aged mice (increased by LPS in aged, but not adult, mice). LPS increased expression of 11 genes (e.g., *C1qa*, *C1qb*, *C3ar1*, *Cfb*, *Il1b*, *Tyrobp*) in the hippocampus of adult mice that were further exacerbated in aged mice. Of these, only expression of *Ccl8* and *Ifit2* were reversed by microglial repopulation. LPS increased expression of 32 genes (e.g., *C3*, *Cd163*, *Mrc1*, *Myd88*) in the hippocampus of aged mice that were not increased in adults. Three of these uniquely regulated genes were prevented by repopulation (*Creb1*, *Daxx*, *Il12a*). Taken together, aged mice had an exaggerated and more comprehensive response to innate immune challenge in the hippocampus compared to adult mice and this response was not reversed by microglial depletion and repopulation.

### Age-associated changes in whole-brain transcription were unaffected by microglial repopulation

**Fig. 7:**
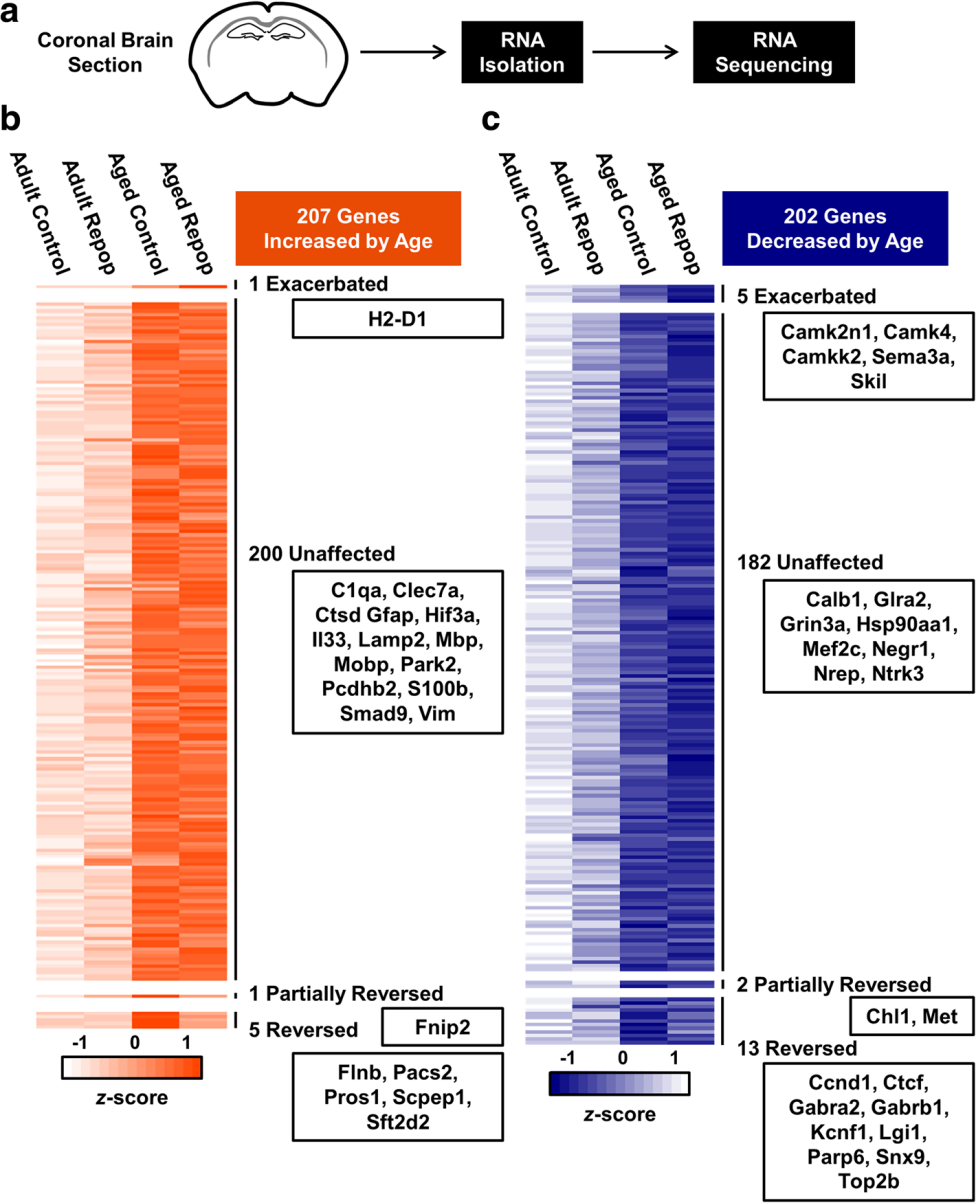
Age-associated changes in whole-brain transcription were unaffected by microglial repopulation. **a** Adult (6–8 weeks old) and aged (16–18 months old) male BALB/c mice were provided diets formulated with vehicle or CSF1R antagonist (PLX5622) for 21 d. After 21 d, all mice were provided vehicle diet for an additional 21 d to allow for repopulation of microglia (Repop). After 21 d of repopulation, a 1-mm coronal brain section was isolated from each brain and whole-tissue RNA was extracted and sequenced. **b** Heat map shows mean expression of 207 genes significantly increased by age (Aged Control vs. Adult Control: *P*_*adj*_ < 0.05). These are divided into categories based on patterns of expression following microglial repopulation: Exacerbated (Aged Repop vs. Aged Control: *P* < 0.05; conserved directionality), Partially Reversed (Aged Repop vs. Aged Control: *P* < 0.05; Aged Repop vs. Adult Control: *P* < 0.05), or Reversed (Aged Repop vs. Aged Control: *P* < 0.05; Aged Repop vs. Adult Control: *P* ≥ 0.05). **c** Heat map showing mean expression of 202 genes significantly decreased by age subdivided as described above. Heat maps are normalized by row and selected genes are shown for each category (*n* = 6 mice / group)

Collectively, we show that microglial repopulation partially reversed age-induced transcriptional changes and lipofuscin accumulation. This, however, was insufficient to reverse exaggerated behavioral and inflammatory responses to innate immune challenge with LPS. Thus, age-associated changes in the brain microenvironment may promote microglial priming independent of repopulation. To address this, we determined the mRNA signature of a coronal brain section, a representative sampling of the whole-brain transcriptome including the cortex, hippocampus, and hypothalamus, in adult and aged mice after microglial depletion and repopulation. Adult and aged mice were administered vehicle or PLX5622 chow for 21 d to deplete microglia. After 21 d, all mice were administered vehicle chow for an additional 21 d to allow for microglial repopulation, after which a coronal brain section was collected, and RNA was extracted and sequenced (Fig. 7a).

Differential expression analysis of whole-brain gene expression between Aged Control and Adult Control groups shows 409 genes significantly affected by age, with increased expression of 207 genes (Fig. 7b) and decreased expression of 202 genes (Fig. 7c). Moreover, these age-associated transcriptional changes persisted despite forced microglial turnover. Microglial repopulation reversed expression of 18 genes (5 increased, 13 decreased; e.g., *Flnb*, *Ctcf*, *Gabra2*, *Gabrb1*, *Parp6*, *Snx*). Only 3 genes were partially reversed, including *Fnip2* (increased by age), *Chl1*, and *Met* (decreased by age). Age-associated decreases in *Camk2n1*, *Camk4*, and *Camkk2* expression, and increase in *H2-D1*, were exacerbated by repopulation. Nonetheless, the majority of age-associated transcriptional changes in the brain were unaffected by microglial repopulation (382 total: 200 increased, 182 decreased). For example, age-associated increases in whole-brain mRNA expression of *C1qa*, *Clec7a*, *Hif3a*, *Il33*, and *Smad9* persisted despite microglial repopulation (Fig. 7b). Similarly, age-associated decreases in expression of *Grin3a*, *Negr1*, *Nrep*, and *Ntrk3* were unaffected by microglial repopulation (Fig. 7c). Notably, age-associated augmentation of astrocyte-related genes, including *Gfap*, *S100b*, and *Vim*, was unaffected by repopulation. Collectively, these data indicate that renewal of microglia following depletion and repopulation did not dramatically influence the whole-brain transcriptional responses to aging in mice.

### Age-associated reactive astrogliosis was microglia-independent

**Fig. 8:**
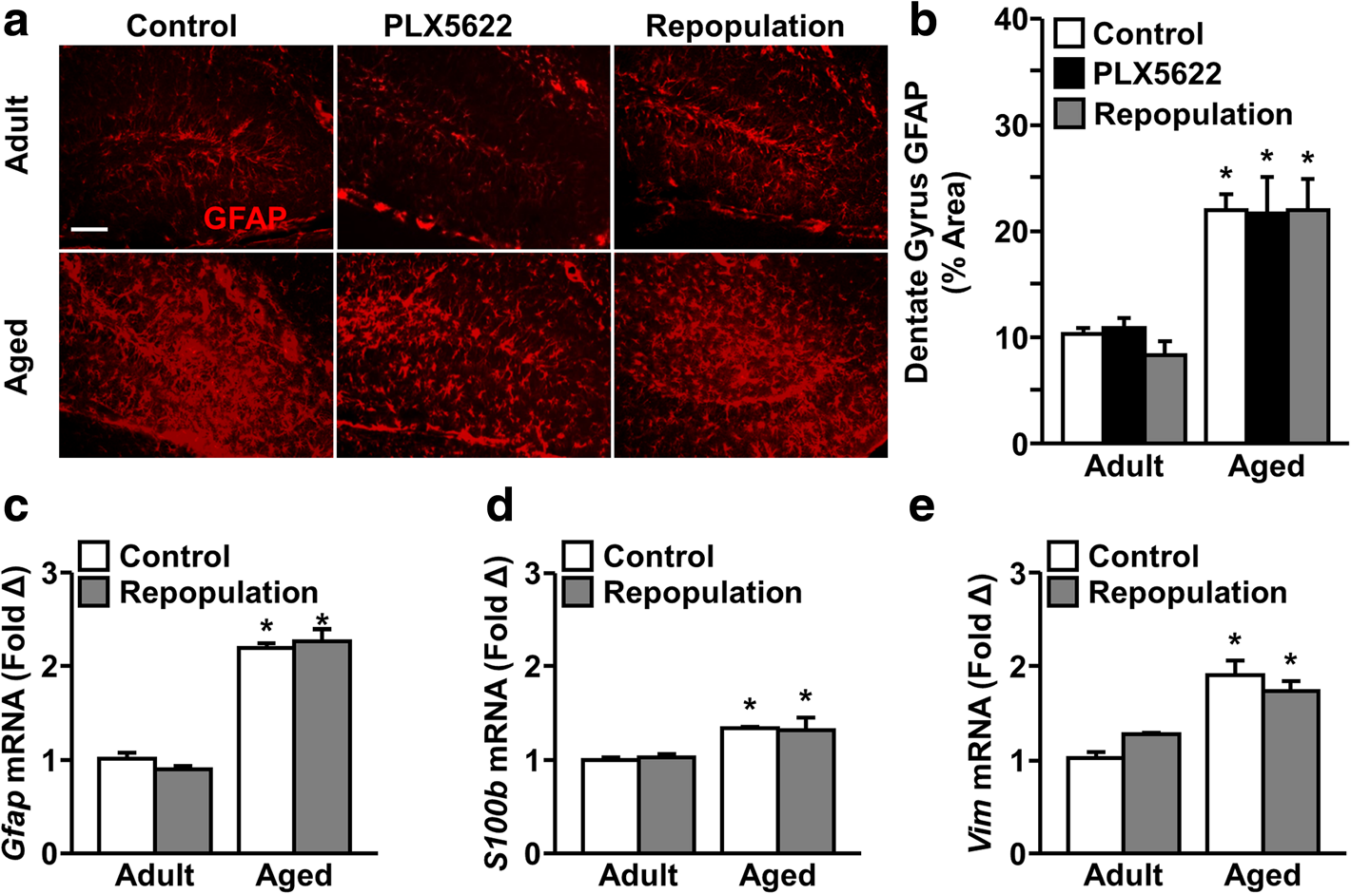
Age-associated reactive astrogliosis was microglia-independent. Adult (6–8 weeks old) and aged (16–18 months old) male BALB/c mice were provided diets formulated with vehicle or CSF1R antagonist (PLX5622) for 21 d. After 21 d, mice were sacrificed or provided vehicle diet for an additional 21 d to allow for repopulation of microglia. After 0 or 21 d of repopulation, hippocampal GFAP reactivity was measured by IHC. **a** Representative GFAP^+^ immunolabeling in the hippocampus of adult and aged mice. Scale bar represents 100 μm. **b** Density of GFAP^+^ astrocytes in the hippocampus with and without microglial depletion and repopulation (*n* = 10–12 mice / group). Similarly, a 1-mm coronal brain section was isolated from mice after 21 d microglial repopulation, RNA isolated, and gene expression analyzed by qPCR. Normalized mRNA expression of *Gfap* (**c**), *S100b* (**d**), and *Vim* (**e**) in the brain (*n* = 3 mice / group). Bars represent the mean ± SEM. Means with * are different from Adult Control (*P* < 0.05)

Several reports indicate that astrocytes become more inflammatory with age [27, 48]. Therefore, we sought to determine the level of reactive astrogliosis in adult and aged mice after microglial depletion and repopulation. Adult and aged BALB/c mice were administered vehicle or PLX5622 chow for 21 d to deplete microglia. After 21 d, all mice were administered vehicle chow for an additional 21 days to allow for microglial repopulation. As expected, GFAP^+^ astrocyte density was increased in the aged hippocampus compared to adults (Fig. 8a, b). There was a significant main effect of age (F(1, 41) = 59.60, *P* < 0.0001) on GFAP^+^ astrocyte density, but not of microglial depletion or repopulation. These findings indicate that the age-associated increase in reactive astrogliosis was independent of microglia.

In a similar study, adult and aged mice were subjected to microglial depletion and repopulation as above. RNA was isolated from a coronal brain section and the expression of genes indicative of reactive astrogliosis was determined **(**Fig. 8c-e). As expected, there was a significant increase in *Gfap* (F(1, 7) = 287.5, *P* < 0.0001), *S100b* (F(1, 7) = 39.68, *P* < 0.001), and *Vim* (F(1, 7) = 44.65, *P* < 0.001) expression in aged mice compared to adults. Moreover, this age-associated increase in mRNA expression was independent of microglial depletion and repopulation. Taken together, these data show that reactive astrogliosis persisted in the aged brain after microglial repopulation.

### Aged brain-conditioned media induces a hyper-inflammatory LPS response in neonatal microglia ex vivo

**Fig. 9:**
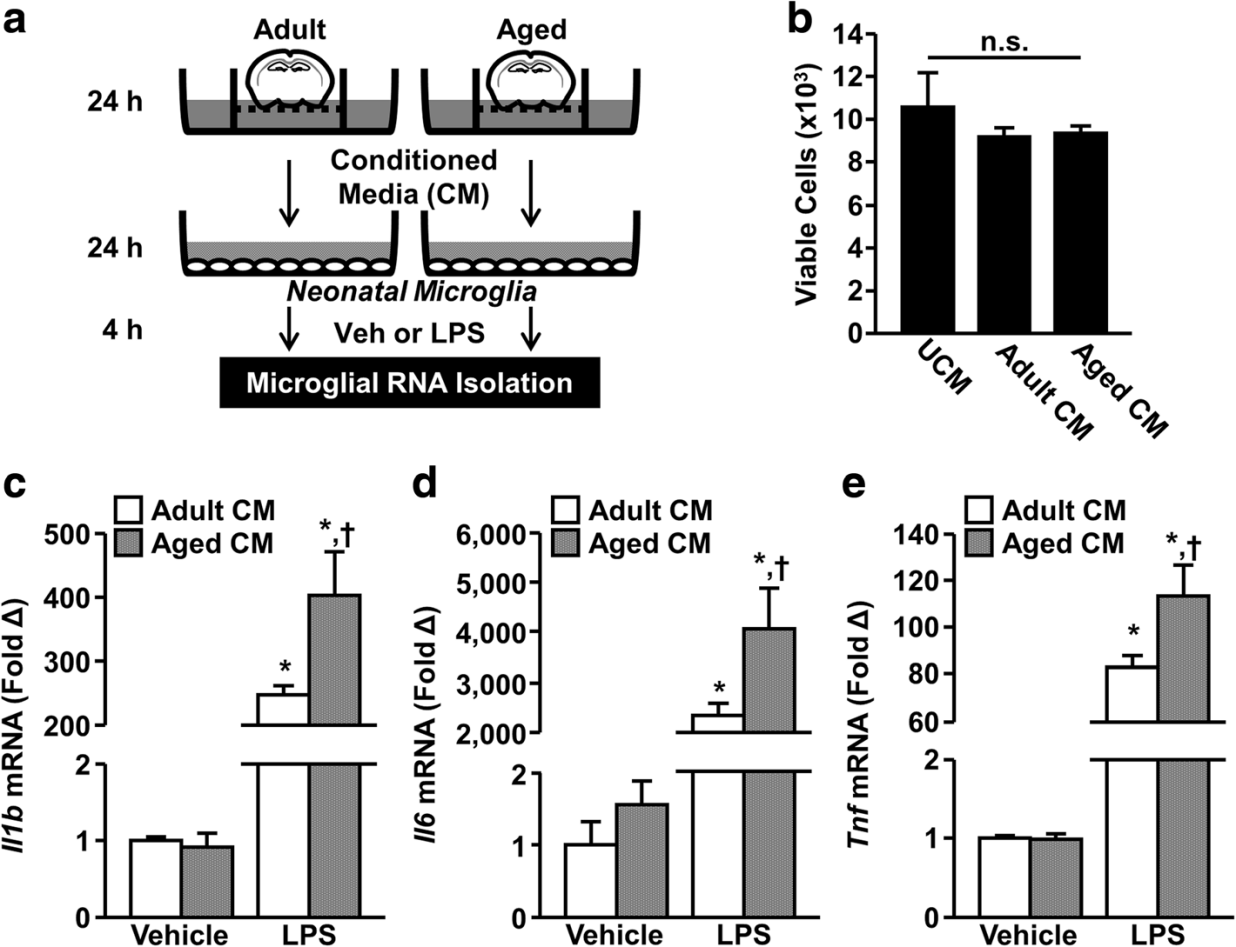
Aged brain-conditioned media induced a hyper-inflammatory LPS response in neonatal microglia ex vivo. **a** Brains were extracted from adult (8–10 weeks old) and aged (20 months old) male BALB/c mice and three 1-mm coronal brain sections from each mouse were used to condition media for 24 h. Conditioned media (CM) was then supplemented with fresh media at a 1:3 ratio. Unconditioned media (UCM) received identical treatment without contact with brain tissue. Primary neonatal microglia were cultured and plated at a density of 1 × 10^4^ cells per well on a 96-well plate, incubated in conditioned medium (adult or aged) for 24 h. Next, primary cultures were stimulated with 1 μg/mL LPS and microglial RNA was collected 4 h later. **b** Viable cell count by condition as determined by MTS assay (*n* = 4 replicates / group). Normalized mRNA expression of *Il1b* (**c**), *Il6* (**d**), and *Tnf* (**e**) in neonatal microglia incubated with adult or aged CM (*n* = 8 replicates / group). Bars represent the mean ± SEM. Means with * are different from Adult CM Vehicle (*P* < 0.05), and means with † are different from treatment-matched Adult CM (*P* < 0.05)

In order to assess the effect of the aged brain microenvironment on the inflammatory signature of microglia, culture media were conditioned with coronal brain sections from adult (8–10 weeks old) or aged (20 months old) BALB/c mice. Again, coronal brain sections were used to represent the global CNS environment. After 24 h, CM was collected and diluted with fresh media. Primary neonatal microglia were then incubated with adult or aged CM for 24 h and stimulated with LPS or vehicle. Microglial RNA was isolated after 4 h and expression of inflammatory cytokines determined (Fig. 9a). It is important to note incubation with CM did not affect microglial viability over this 24-h period (Fig. 9b). As expected, there was a significant main effect of LPS on expression of *Il1b* (F(1, 28) = 81.6, *P* < 0.0001), *Il6* (F(1, 28) = 57.7, *P* < 0.0001), and *Tnf* (F(1, 28) = 176.5, *P* < 0.0001) mRNA expression (Fig. 9c-e). Moreover, microglia incubated with aged CM exhibited increased expression of *Il1b*, *Il6*, and *Tnf* mRNA in response to LPS compared to adult CM (*P* < 0.05 for all). Taken together, these data show soluble signals from the aged brain cause microglia to exhibit exaggerated inflammatory responses to LPS stimulation.

## Discussion

Our primary objective was to test if age-associated microglial priming and hyperactivity to immune challenge is reversed by promoting microglial turnover by CSF1R antagonism. We depleted microglia from both adult and aged BALB/c mice and subsequently allowed new microglia to fully repopulate the brain. We provide novel evidence that forcing microglial turnover in aged mice reversed age-associated increases in intracellular lipofuscin accumulation and CD68^+^ lysosome size. Furthermore, microglia-specific RNA sequencing revealed a 511-gene signature differentiating aged microglia from adult microglia. Of these, expression of 127 genes was reversed by microglial repopulation. Despite this partial reversal of the aged microglial transcriptome, LPS challenge still elicited exaggerated sickness behavior and CNS inflammation in aged mice regardless of microglial repopulation. Thus, we provide novel evidence that the aged brain microenvironment may cause microglial priming. For instance, the whole-brain transcriptional signature of aging was unaffected by repopulation of microglia. Furthermore, soluble factors from the aged microenvironment prime neonatal microglia to LPS ex vivo. While there may be microglia-intrinsic aspects of aging that can be reversed by forced microglial turnover, the brain microenvironment re-establishes the pro-inflammatory and primed profile characteristic of aging.

An important finding is that microglia present after forced turnover in aged mice were different from those in aged controls. “New” microglia had reduced intracellular lipofuscin and smaller CD68^+^ lysosomes. A previous report showed aged microglia had increased lipofuscin burden and larger CD68^+^ lysosomes due to homeostatic clearance of myelin debris throughout the lifespan [60]. Furthermore, previous reports indicate that microglial repopulation reverses the proportion of CD68^+^ microglia in aged mice [21]. We extend these findings to show that, while all microglia contain CD68^+^ lysosomes, confocal microscopy revealed a restoration of age-associated CD68^+^ lysosome enlargement. Additionally, our findings indicate that the lipid-laden phenotype observed in aged microglia was reversed by microglial depletion and subsequent proliferation. It is important to highlight that these changes were observed with only 80–85% depletion of microglia. Collectively, these findings suggest that promoting microglial turnover restores age-related dysfunction in lysosomal structure and intracellular lipofuscin accumulation.

In addition to structural alterations in repopulated aged microglia, repopulated microglia had an intermediate RNA signature. For instance, of the 511 genes differentially expressed by age, 127 were reversed by repopulation. These include various scavenging receptors implicated in Alzheimer’s disease pathogenesis that were increased with age and reversed by microglial repopulation (e.g., *A2m*, *Apoe*, *Olr1*, *Sorl1*). TGF-β family ligands, *Bmp6* and *Tgfb*, were increased with age and restored by microglial repopulation. Microglial repopulation also reversed age-associated decreases in expression in mRNA encoding suppressor of cytokine signaling-3 (*Socs3*), which inhibits inflammatory cytokine and chemokine production and may reflect a mechanism for elevated cytokine production in aged microglia. Age-associated differential expression of 58 genes (53 increased, 5 decreased) was partially reversed by microglial repopulation, such that expression moved towards adult control levels but remained significantly different. For instance, age-associated alterations in genes related to innate-immune sensing (*Lyz2*, *Tlr5*), chemokines (*Ccr1*), and cytokine signaling (*Tgfbr3*) were partially reversed. Collectively, these findings suggest that forced turnover of aged microglia was sufficient to restore lipofuscin accumulation and lysosome dysfunction, and to partially reverse the aged microglial transcriptional signature.

Despite the intermediate RNA profile described above, 307 of 511 differentially regulated genes with age were unaffected by microglial repopulation. Unaffected genes included the pro-inflammatory cytokine *Il1b* and the MHC class II component *H2-Eb1*, which are implicated in age-associated microglial priming [24, 29, 68]. Notably, genes associated with a conserved neurodegenerative microglial phenotype were altered with age and either exacerbated (*Axl*) or unaffected by microglial repopulation (*Clec7a*, *Itgax*) [38]. Despite divergent aging signatures in human and mouse microglia, we found conserved genes (*Cxcr4*, *Tnfaip2*) were unaffected by repopulation [25]. Despite partial or full reversal of 185 genes, pathway analysis showed little benefit of repopulation. For example, differential gene expression was consistent with age-associated increases in NF-κB signaling, production of NO/ROS by macrophages, and elevated IFNγ, IFNα, IL-1β and TNF signaling in microglia. This is consistent with previous reports of elevated baseline inflammation and ROS production in aged microglia [70]. Furthermore, elevated STAT3 and increased cell motility/movement were previously described in mice and humans, respectively [25, 30]. Neither these pathways nor overall ontological classifications of genes regulated by age were influenced by microglial repopulation. These findings are consistent with PCA, suggesting there is an overall effect of age on transcription that is not fully reversed by repopulation. Collectively, microglial repopulation reverses age-associated increases in intracellular lipid accumulation, but incompletely restores the aged microglial transcriptome.

Intermediate restoration of the microglial mRNA profile in aged mice was insufficient to prevent age-associated exacerbation of sickness behavior or amplified neuroinflammation following peripheral LPS challenge. Aged mice, regardless of microglial repopulation, had prolonged and exaggerated sickness behavior following LPS challenge compared to adult controls. Analysis of RNA copy number in the hippocampus confirmed and extended previous findings that aged mice have exaggerated neuroinflammatory responses to LPS [29, 46]. Here, we extend the previous literature to differentiate the aged LPS response into two categories: genes increased by LPS in adult mice but *exacerbated* by aging and genes *uniquely* increased by LPS in aged mice but not in adults. Neither genes exacerbated nor uniquely regulated in aged mice following peripheral LPS were dramatically influenced by microglial repopulation. This is consistent with recent findings reported by Elmore et al. in which whole-brain inflammatory gene expression remained exaggerated in aged mice 6 h after peripheral LPS administration regardless of microglial repopulation. Notably, we found that numerous complement components (*C1qa, C1qb, C3ar1*, *Cfb*) and the inflammatory cytokine *Il1b* were exacerbated by age and unaffected by repopulation. Furthermore, the aged LPS response was more comprehensive than adult mice, and included genes related to extracellular remodeling (*Mmp9*), pathogen recognition (*Tlr2, Tlr7*), and interferon responsiveness (*Ifit5*). Of the 43 genes comprising the LPS signature in aged mice, only 5 were reversed by microglial repopulation. Thus, microglial repopulation was insufficient to reverse age-associated microglial priming to peripheral immune challenge.

Another relevant point of discussion is that the aged microenvironment was unaffected by microglial renewal and likely influences repopulating microglia. It is important to note that the inflammatory signature of the aged brain is conserved throughout the brain and across species [40]. Thus, the persistence of an inflammatory/damaged microenvironment in the aged brain may explain why microglial repopulation in mice was insufficient to reverse age-associated exacerbation of sickness behavior and neuroinflammation following LPS challenge. In support of this, microglial depletion and repopulation reduced the level of lipofuscin in aged microglia, but not in the aged neurons. Thus, neurons remained lipid-laden in the aged brain, which is associated with elevated oxidative stress [65]. Moreover, the aging mRNA signature in a coronal brain section (10% microglia) was unaffected by microglial depletion and repopulation. Genes related to astrocyte reactivity (*Gfap*, *S100b*, *Vim*), neurotrophic/growth factors (*Negr1*, *Nrep*, *Ntrk3*), cell death (*Anxa4*), neurotransmitter signaling (*Grin3a*, *Glra2*), and myelin (*Mbp*, *Mobp*) were all dysregulated with age and unaffected by microglial repopulation. It is important to highlight that these findings differ from a recent report that microglial repopulation restores age-associated dysregulation in genes associated with neuronal health [21]. Elmore et al. found age-associated increases in whole-brain inflammatory gene expression was unaffected by microglial repopulation. This was in contrast to genes related to cytoskeletal rearrangement and synaptogenesis, which were restored with microglial renewal in aged C57BL/6 mice. Of the 820 transcripts regulated by age in whole-brain RNA between Elmore et al. and our dataset, only 29 are shared in both analyses. This may explain the discrepancies between our conclusions regarding the overall benefit of microglial repopulation with age. Nonetheless, we both observed that microglial repopulation was insufficient to prevent immune and inflammatory priming to peripheral LPS. We further characterized the microglia-specific transcriptome and found an intermediate expression profile, with restoration of some, but not all, inflammatory genes. We interpret these findings to suggest that some microglia-intrinsic aspects of microglial aging can be reversed by repopulation, but overall they become primed as they repopulate in the aged brain.

Microglial repopulation did not reverse evidence of age-induced astrogliosis, which may play a role in priming repopulating microglia. Elevated *Gfap*, *S100b*, and *Vim* expression is associated with reactive astrogliosis in the aged brain [11, 48, 50, 74], all of which were increased with age in whole-brain RNA regardless of mciroglial repopulation. Consistent with these results, we detected higher GFAP mRNA and protein expression in the hippocampus. Others report astrocytes in the aged brain have an mRNA profile associated with dysfunction (i.e., less supportive of growth, repair, and regulation) [59]. This is relevant because recent evidence shows that microglia-astrocyte communication helps to resolve microglial activation after peripheral immune challenge [46, 48]. Furthermore, astrocytes dynamically respond to environmental cytokines, including IL-1, TNFα, and C1q, and this neurotoxic/inflammatory phenotype may persist independent of microglial repopulation [39]. Here, we found neonatal microglia cultured with conditioned media from aged coronal brain sections had increased responsiveness to direct LPS stimulation and had higher levels of *Il1b*, *Il6* and *Tnf* compared to those cultured with adult conditioned media, a response consistent with microglial priming [45, 47]. Thus, either the presence or absence of soluble factors from the aged brain causes microglia to be primed to LPS challenge. We do not, however, know which factor or factors are critical for this re-direction of the microglial LPS response. Collectively, these data indicate age-associated microglial priming is not intrinsic to microglia; rather, microglia develop a primed phenotype in response to elevated inflammation, oxidation, or damage present in the aged brain [40].

Despite persistence of immune priming, microglial turnover may have some benefits. Elmore et al. report microglial repopulation restored age-associated cognitive decline and synapse loss [21]. Furthermore, the benefit of microglial repopulation may be more profound in contexts with spatially or temporally restricted injury, in contrast to advanced age where CNS damage is altered progressively throughout the brain. For example, microglial turnover following inducible hippocampal lesion and neuron death ameliorated chronic microgliosis, leukocyte infiltration, and inflammatory gene expression [55]. These experiments were completed using adult and otherwise healthy mice. We also have data that forced microglial turnover in a model of stress (e.g., repeated social defeat) reversed microglial priming to LPS challenge ex vivo and in vivo [71]. These data support the conclusion that whether forced turnover of microglia is sufficient to alter responses to subsequent stimuli is context-dependent. In the context of advanced age, our results indicate that the aged CNS microenvironment plays a major role in the development of the pro-inflammatory profile of microglia. This may have significant ramifications for therapeutic approaches looking to replace microglia in the aged, injured, or diseased brain. Based on our current findings, microenvironmental consequences of aging or disease are likely to influence the development of repopulating microglia towards their original compromised phenotype.

There may be specific functional benefits to microglial physiology with depletion and repopulation in the aged brain. As discussed, microglial repopulation reduced CD68 expression, cleared lipofuscin, and partially restored the microglial RNA signature. Because microglia have myriad proposed functions beyond host-defense, including support of neurodegeneration and dynamic phagocytosis of synapses, it is plausible there is a direct benefit of repopulation. We, however, did not systemically test each proposed function of microglia here. Rather, our goal was to determine if microglial priming and exacerbated immune reactivity to LPS challenge in aged mice could be reversed. Priming and immune reactivity in the microglia of aged mice was not reversed by forcing microglial turnover. Nonetheless, we provide significant findings that indicate the local microenvironment of microglia contributes strongly to their phenotype in the brain.

In summary, we provide original and compelling evidence that microglia can be removed and repopulated in the aged brain. As such, the lipid-laden aged phenotype was reversed by forced microglial turnover. This turnover also resulted in partial reversal of the aged microglia RNA signature. Nonetheless, priming and immune-reactive RNA signatures were still detected after repopulation of aged microglia. As a result, LPS challenge still induced an exaggerated microglial inflammatory response in the aged brain. To explain why these new microglia remain primed, we provide RNA sequencing of whole-brain tissue with clear evidence of “inflammaging” that was not restored by microglial turnover. Indeed, conditioned media generated from the brain of aged mice was sufficient to recreate the primed response in developing neonatal microglia ex vivo. Thus, we conclude that the forced renewal of microglia in the aged brain cannot overcome the environmental cues of the inflamed brain as they repopulate.

## Additional file

Additional file 1: Adult (6–8 weeks old) and aged (16–18 weeks old) male BALB/c mice were provided diets formulated with vehicle or CSF1R antagonist (PLX5622) for 21 d. After 21 d, all mice were provided vehicle diet for an additional 21 d to allow for repopulation of microglia (Repop). After 21 d of repopulation, a 1-mm coronal brain section was isolated from each brain, Percoll-enriched microglia were sorted from the remaining tissue, and RNA was collected and sequenced. Counts were normalized using DESeq2 in R. Three CSV files are provided that describe sample groups (sample_key.csv), normalized microglia counts (microglia_norm_counts.csv), and normalized coronal brain section counts (brain_norm_counts.csv). (ZIP 5420 kb)
